# Incidental Intraoperative Finding of Bilateral Lipoma Arborescens in a Patient Undergoing Bilateral Total Knee Replacement

**DOI:** 10.7759/cureus.23692

**Published:** 2022-03-31

**Authors:** Abdulhakim H Alshehri, Hamad Alshahrani, Omar A Salem, Doaa A Alfaifi

**Affiliations:** 1 Orthopedic Surgery, King Fahad Military Medical Complex, Dammam, SAU; 2 Orthopedic Surgery, King Fahad Specialist Hospital, Dammam, SAU; 3 Orthopedics, King Fahad Specialist Hospital, Dammam, SAU; 4 Pathology, King Fahad Specialist Hospital, Dammam, SAU

**Keywords:** synovial hyperplasia, chronic synovitis, synovial tumor, knee, : lipoma arborescens

## Abstract

Lipoma arborescens (villous lipomatous proliferation of the synovial membrane) is a rare, benign articular lesion characterized by diffuse villous proliferation of the synovium with mature fat substitution of the sub-synovial connective tissue. The etiology of this condition still remains unknown. It represents part of differential diagnosis for a progressive swollen knee with chronic pain secondary to synovial proliferative disorders with or without associations with osteoarthritis. We report an intraoperative finding of lipoma arborescence in a patient who underwent bilateral primary total knee replacement secondary to advanced osteoarthritis.

## Introduction

Knee pain and swelling are common presentations in orthopedics and rheumatology. Being chronic in nature would raise the suspicion of proliferative synovial disorders [1]. Tumors of the synovium are rare, and most of them are benign. Lipoma arborescence (LA) is a rare benign synovial tumor described first in 1904 by Hoffa et al. as a type of inflammatory hyperplasia of the adipose tissue of the knee [2,3]. Most of the reported cases presented with a progressive recurrent effusion with or without pain [3,4]. Lipoma arborescens has been categorized in the literature as primary and secondary [5]. The secondary type is more common and usually associated with chronic irritation, osteoarthritis, diabetes mellitus, rheumatoid arthritis, synovitis, and meniscal injury. The primary type is less common and idiopathic in nature, usually affecting younger patients in the first two decades of life [6]. Lipoma arborescens has a higher prevalence in the male population and has been reported in patients from ages nine to 66 years [7]. It commonly affects the knee joint, specifically the suprapatellar pouch [1,4,8], but there have been reported involvement of other joints, including the hip [9], shoulder [10], wrist [11], and elbow [12]. Lipoma arborescens is usually mono-articular, but cases of bilateral involvement of the knees have also been published [1,3,5,13]. Histological examination of the specimen reveals villiform fatty tissue covered by slightly thickened synovium characteristic of lipoma arborescens [14]. Degenerative or inflammatory arthritis is quite often reported among patients with LA, ranging from early arthritis up to advanced knee osteoarthritis, as reported in our case [3,15,16]. Although the consequences of LA have not been defined yet, early detection and treatment may prevent further progression [17].

MRI is the best diagnostic modality in patients suspected of LA as it offers a clear characteristic entity of LA on fat suppression images or short TI inversion recovery (STIR) sequence [18]. The MRI feature of LA comprises multiple villous lipomatous synovial proliferations and a 'frond-like' projection together with the multilobulated synovial proliferation of fat signal intensity [19]. Other MRI findings include joint effusion degenerative changes with meniscal tears, synovial cysts, bony erosion, and chondromatosis [20]. Cases have been described with abnormality or absence of the meniscus [21]. The gross clinical picture of the lipoma arborescens is a tree-like appearance with multilobulated fatty projections [4].

## Case presentation

**Figure 1:**
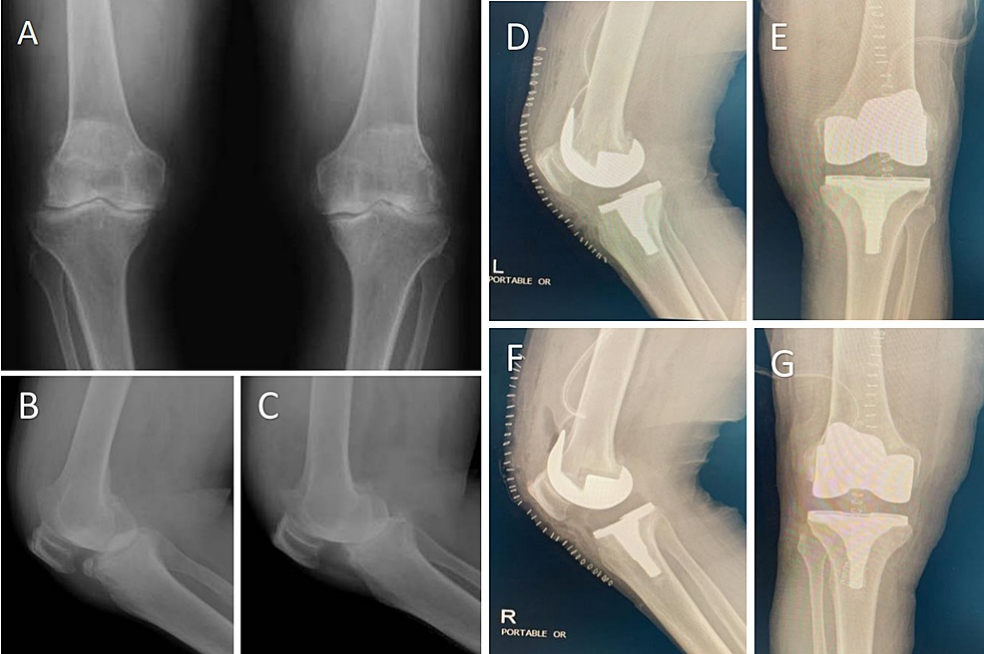
Pre-operative X-ray and post bilateral total knee arthroplasty X-ray Pre-operative X-ray shows tri-compartments advanced osteoarthritic changes at both knees manifested by significant joint space loss, subchondral sclerosis, and marginal osteophytes formation. Bilateral moderate effusions are seen. Mild genu varus deformity was noted on both sides (A-C). Post bilateral total knee arthroplasty X-ray study shows intact hardware appearance and anatomic alignment (D-G).

A 64-year-old man known to have hypertension presented to an arthroplasty clinic, complaining of bilateral knee pain that started six years ago with progressive pain aggravated by movement and affecting his daily activity. He was initially treated in another hospital by conservative modalities using nonsteroidal anti-inflammatory drugs (NSAID), physiotherapy, and multiple intra-articular cortisone injections. The patient was found to have a BMI of 32, bilateral mild flexible varus deformity of the knees, swelling, no inflammatory signs, suprapatellar effusion, and mild restricted range of motion. Additionally, he was found to have multiple Heberden's nodes involving proximal interphalangeal (PIP) and distal interphalangeal (DIP) joints of both hand digits, advanced osteoarthritis, and normal screening blood tests (erythrocyte sedimentation rate [ESR], C-reactive protein [CRP], white blood cells [WBC]). Plain X-rays of both knees are presented in Figures 1A-C. The patient was elected for bilateral total knee replacement (see Figures 1D-G).

**Figure 2:**
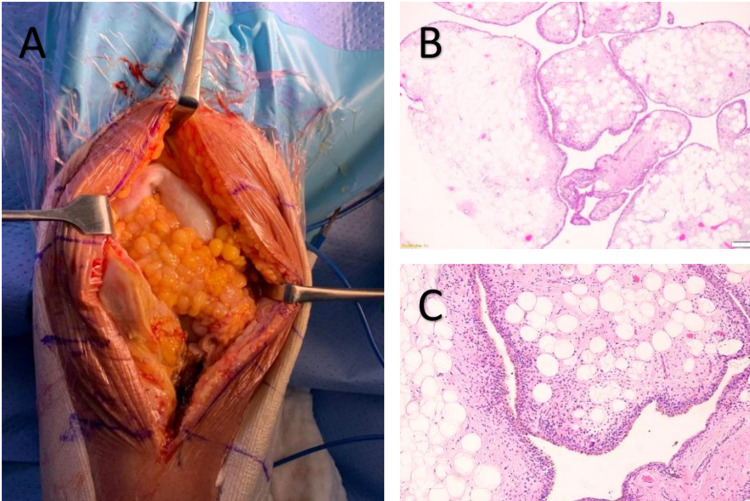
Intra-operative image and images of histological stains Intra-operative image shows a large intra-articular fatty tumorous tissue (A). Hematoxylin and eosin stain (4x magnification) shows multiple hypertrophic villi filled with fat cells (B), hematoxylin and eosin stain (40x magnification) shows multiple villi lined with synovial cells, filled with fat cells, and few inflammatory cells (C).

Intraoperatively, there were findings of multilobulated fatty projections occupying the suprapatellar region (Figure 2A), thickened synovium with an abundant amount of synovial fluids that came out during arthrotomy, friable surrounding tissues, and hypoplastic popliteal oblique ligament of both knees. A histopathology sample was sent to the lab, and he was referred for a rheumatology opinion. Further blood tests, including rheumatoid factor, human leukocyte antigen B27 (HLA-B27), anti-cyclic citrullinated peptide (CCP), were all negative. The diagnosis of bilateral lipoma arborescens of the knees was made based on a histopathology report. Total resection of the lipoma was done combined with synovectomy.

Grossly, the specimens consist of multiple pieces of frond-like fatty tissue 5 cm in size bilaterally. There were no areas of necrosis or hemorrhage. Microscopic examination showed mature adipocytes forming the expansive villous lesion lined by synovial cells without atypical features with a central fibrovascular axis and discrete lymphoplasmacytic infiltrate (Figures 2B-C). 

## Discussion

The etiology of this condition still remains unknown. It represents part of differential diagnosis for a progressive swollen knee with chronic pain secondary to synovial proliferative disorders with or without associations with osteoarthritis. Tumors of the synovium are rare, and most of them are benign. Lipoma arborescence is a rare benign synovial tumor described first in 1904 by Hoffa et al. as a type of inflammatory hyperplasia of the adipose tissue of the knee [2,3]. Most of the reported cases presented with a progressive recurrent effusion with or without pain [3,4]. Degenerative or inflammatory arthritis is quite often reported among patients with LA, ranging from early arthritis up to advanced knee osteoarthritis, as reported in our case [3,15,16]. Although the consequences of LA have not been defined yet, early detection and treatment may prevent further progression [17].

In comparison to our case, we had an intraoperative finding of marked friability of the joint surrounding tissues and hypoplastic popliteal oblique ligament bilaterally along with a presentation of knee pain with slowly progressive swelling frequently described as well recurrent effusion with or without mechanical symptoms. Synovectomy, either arthroscopic or open, is the best curative modality reported that imparts total resection of the entire mass and has been acknowledged to provide long-lasting relief of the associated symptoms [1,22]. Although the consequences of LA remain unknown, the development of early osteoarthritis as a complication of delayed synovectomy has been conveyed [22].

## Conclusions

Lipoma arborescens (villous lipomatous proliferation of the synovial membrane) is a rare, benign articular lesion characterized by diffuse villous proliferation of the synovium with mature fat substitution of the sub-synovial connective tissue. The etiology of this condition still remains unknown. It represents part of differential diagnosis for a progressive swollen knee with chronic pain secondary to synovial proliferative disorders with or without associations with osteoarthritis. LA is a rare cause of knee pain and swelling. Treatment varies and depends on the presentation. We report an intraoperative finding of lipoma arborescence in a patient who underwent bilateral primary total knee replacement secondary to advanced osteoarthritis. 
